# Evaluation of the Effects of Drying Techniques on the Physical and Nutritional Characteristics of Cricket (*Gryllus bimaculatus*) Powder for Use as Animal Feedstuff

**DOI:** 10.3390/insects16080814

**Published:** 2025-08-06

**Authors:** Warin Puangsap, Padsakorn Pootthachaya, Mutyarsih Oryza, Anusorn Cherdthong, Vibuntita Chankitisakul, Bundit Tengjaroensakul, Pheeraphong Phaengphairee, Sawitree Wongtangtintharn

**Affiliations:** 1 Department of Veterinary Public Health, Faculty of Veterinary Medicine, Khon Kaen University, Khon Kaen 40002, Thailand; warin.paun@kkumail.com (W.P.); btengjar@kku.ac.th (B.T.); 2 Department of Animal Science, Faculty of Agriculture, Khon Kaen University, Khon Kaen 40002, Thailand; padsakornp@kkumail.com (P.P.); anusornc@kku.ac.th (A.C.); vibuch@kku.ac.th (V.C.); perpan@kku.ac.th (P.P.); 3 School of Animal Technology and Innovation, Institute of Agricultural Technology, Suranaree University of Technology, Nakhon Ratchasima 30000, Thailand; mutyaoryzasativa94@gmail.com

**Keywords:** drying method, physical properties, nutritional composition, protein source

## Abstract

This study aimed to evaluate the effects of three drying methods, namely sun drying, microwave–vacuum drying, and hot-air-oven drying, on the physical and nutritional properties of cricket powder for use in poultry feed. The results showed that the drying method significantly affected color parameters (L*, a*, and b*; *p* < 0.05), and particle size distribution at 850 µm and 250 µm (*p* = 0.04 and *p* = 0.02, respectively). Microwave–vacuum drying produced the lightest powder with a higher proportion of coarse particles, while sun drying resulted in a darker color and greater particle retention at 850 µm. Hot-air-oven drying yielded the lowest moisture content (1.99%) and the highest gross energy (6126.43 kcal/kg), with no significant differences observed in crude protein (*p* = 0.61), ether extract (*p* = 0.08), crude fiber (*p* = 0.14), ash (*p* = 0.22), or amino acid profiles (*p* > 0.05). These findings indicate that all drying methods preserved the nutritional value of cricket powder, and based on the overall results, hot-air-oven drying is the most suitable method for producing high-quality cricket meal with optimal physical properties and feed value, while also providing a practical balance between drying efficiency and cost.

## Simple Summary

This study investigated the effects of different drying methods, including sun drying, microwave–vacuum drying, and hot-air-oven drying, on the quality of cricket powder for use as a poultry feed ingredient. The results showed that while the drying method influenced physical characteristics such as color, particle size, and flowability, it had no statistically significantly impact on the protein content or amino acid profile. Among the methods tested, hot-air-oven drying was the most suitable, yielding the lowest moisture content while optimizing both nutritional value and physical properties. These findings support the potential use of cricket powder as a sustainable and nutritionally valuable alternative protein source in animal diets.

## 1. Introduction

The global population is projected to exceed 9 billion by 2050, driving a substantial increase in the demand for animal-based protein, which is estimated to rise by 70–80% [1]. This creates an urgent need for sustainable protein sources to support global food security [2]. The livestock industry faces challenges in meeting the rising demand for animal products, which often surpasses the existing production capacity. Furthermore, reliance on traditional protein sources is becoming increasingly unsustainable, highlighting the need to explore alternative raw materials [3]. Conventional protein sources such as soybean meal and fishmeal are not only costly but also largely imported, thereby contributing to higher production expenses [4]. Additionally, these protein sources directly compete with human food supply chains, further intensifying resource constraints [5]. Consequently, the livestock sector is increasingly exploring cost-effective and nutritionally viable alternative protein sources, such as edible insects.

Edible insects are increasingly recognized as a sustainable and efficient alternative protein source, offering a promising solution to the growing challenges of global food and nutritional security for both humans and animals [6]. In addition to their high protein content, edible insects are also rich in essential fatty acids, minerals, and micronutrients that are critical for animal growth and development [7]. Among these, crickets (*Gryllus bimaculatus*) have gained attention as an economically important species with a high potential for inclusion in animal feed. They possess a remarkable nutritional profile, featuring high protein and fat contents [7], and they are also a valuable source of essential amino acids and a diverse array of mineral elements [8]. The utilization of crickets as an alternative protein source in animal feed is gaining attention, particularly as a replacement for fishmeal and soybean meal in the diets of poultry, swine, and ruminants. For instance, Fisher et al. [9] reported that including up to 20% cricket meal in broiler diets had no adverse effects on growth performance, organ indices, or meat quality. Boontiam et al. [10] reported that crickets can successfully replace soybean meal and fishmeal in pig diets without causing adverse effects, while also enhancing growth performance and intestinal morphology. Similarly, Phesatcha et al. [11] found that crickets could be used as a protein supplement in ruminant diets to improve propionate production in the rumen, reduce protozoal populations, and lower methane emissions.

However, to effectively incorporate crickets into animal feed, they must first be processed into powder form. This transformation enhances their digestibility, ensures formulation consistency, and extends their shelf life [6]. Importantly, the physical and chemical properties of the processed cricket powder are key factors in determining the overall efficiency and cost-effectiveness of animal feed production. The drying method used during processing has a significant impact on maintaining the powder’s nutritional value and influencing the final product’s physical characteristics [12]. As mentioned by Huang et al. [13], the particle size and drying methods utilized can affect the digestibility of crickets’ nutrients and energy. Moreover, the utilization of crickets as animal feed is still currently limited, and only a few studies have specifically investigated the impact of different drying methods on the particle size and chemical composition of cricket powder in animal feed applications [9,10]. Previous research has largely focused on the overall nutritional benefits of insects, but insufficient attention has been given to how specific drying techniques impact the physical and nutritional characteristics relevant to feed formulation and efficiency [11].

As mentioned above, it is necessary to develop standardized criteria for the utilization of cricket powder as an animal feed ingredient, with a clear focus on both its physical characteristics and nutritional value. Currently, Thailand lacks official guidelines or quality standards for the procurement and evaluation of cricket powder. This regulatory gap presents significant challenges for feed manufacturers in evaluating and selecting cricket powder as a raw material. The variability and lack of regulation in the quality and consistency of commercially available cricket powder further complicate its adoption in feed formulation. Establishing standardized criteria would enable feed producers to ensure the quality of their raw materials and promote the consistent production of high-quality cricket powder.

Therefore, the objective of this study was to determine the most suitable drying method for producing high-quality cricket powder as a sustainable protein ingredient in animal feed. Three drying techniques, sun drying, microwave–vacuum drying, and hot-air-oven drying, were compared, evaluating their effects on the physical properties influencing feed handling and storage efficiency, as well as the chemical properties of cricket powder, including its nutritional value and potential impact on feed quality.

## 2. Materials and Methods

### 2.1. Sample Collection

Forty-two-day-old field crickets (*Gryllus bimaculatus*) were purchased from the Ban Saen Tor Cricket Farmers Community Enterprise, Nam Phong District, Khon Kaen Province, Thailand (Latitude 16°36′23.76″ N, Longitude 102°56′47.72″ E). The crickets were farmed in accordance with Thailand’s Good Agricultural Practices system. In preparing the crickets, 9 kg of fresh samples was washed with clean water and subsequently utilized to investigate the effects of different drying methods. The crickets were randomly assigned to three drying treatment methods: sun drying, microwave–vacuum drying, and hot-air-oven drying, in a completely randomized design (CRD). Each treatment group consisted of 3 kg of crickets, divided into three replications of 1 kg.

The drying process was conducted according to the method described by Pornsuwan et al. [14]. For sun drying, a 3 kg sample of crickets was placed in a tray and dried in a solar dome (6.0 m × 8.2 m in area, with a central height of approximately 3.8 m and side wall height of 2.2 m). The drying process continued for 48 h under typical tropical conditions, with the internal temperature reaching up to 60 °C during peak sunlight hours. For microwave–vacuum drying, a rotary microwave–vacuum dryer (March Cool Industry Co., Ltd., Bangkok, Thailand) was used. The samples were divided into three batches of 1 kg each and dried sequentially for 3 min at 800 watts per batch, with the drying time for the three sets totaling 9 min, while ensuring that the temperature did not exceed 60 °C. For hot-air-oven drying, the oven (30-UF1060, Memmert GmbH + Co. KG, Schwabach, Germany) was preheated for 15 min. Then, 3 kg of crickets was spread evenly on a tray and dried at 60 °C for 48 h, or until the moisture content was reduced to below 10%. After drying, all samples were ground using a fine grinder (Mill powder grinder (Model RT-04), Mill Powder Tech Co., Ltd. (MPT), Tainan, Taiwan) to enable them to pass through a 1.0 mm sieve and were subsequently prepared for further analysis.

### 2.2. Physical Characteristic Measurement

#### 2.2.1. Bulk Density

The bulk density of the cricket powder was measured by filling a 1000 mL cylinder with samples from each group of cricket powder. The filled cylinder was then weighed and the bulk density was calculated. Each sample was analyzed in 15 replicates, following the method of Tanpong et al. [15].

#### 2.2.2. Angle of Repose

The angle of repose of the cricket powder was measured by pouring 100 g of each sample from a predetermined height through a funnel onto a flat surface. The funnel’s opening was positioned at a fixed distance above the surface, and a protractor was used to measure the angle on both the left and right sides of the sample, following the method of Tanpong et al. [15]. Each sample was analyzed in 15 replicates.

#### 2.2.3. Color Parameter

The color of the cricket was determined using a Chroma Meter (CR-410 Series, Konica Minolta Sensing Inc., Tokyo, Japan) with CIELAB coordinates (L*, a*, b*). In this color space, L* indicates lightness (with higher values representing lighter colors and lower values representing darker colors), a* indicates redness (with higher values representing redder colors and lower values representing greener colors), and b* indicates yellowness (with higher values representing yellower colors and lower values representing bluer colors), following the method of Purschke et al. [16]. Each sample was analyzed in 10 replicates. Furthermore, the browning index (BI) at each measuring point was calculated using the following equation [17]:

BI = 100 × [(X − 0.31)/0.17]

where X = [(a* + 1.75 × L*)/(5.645 × L* + a* − 3.012 × b*)]

#### 2.2.4. Particle Size and Distribution

The particle size distribution was measured using an electric sieve shaker, which consisted of meshes sized 20, 40, 60, 80, and 100 mesh (Laboratory Test Sieve Series, Endecott’s Ltd., London, UK). For each group, 100 g of cricket powder was used, and the shaker was operated for 20 min. Each sample was measured five times. After the shaking process, the weight of each layer was recorded to calculate the percentage of retention and determine particle movement, following the method described by Oryza et al. [18], as follows:

Retain (%) = [Total sample weight in the sieve/Total weight of sample] × 100

Passing (%) = 100 − Retain (%)

#### 2.2.5. Microscopic Characterization

The morphology structures of crickets from each sample group were examined using a stereo microscope (JSZ5B, Novel Optics Co., Ltd., Beijing, China) at magnifications of 20× and 60×, following the methods described by Tanpong et al. [15].

### 2.3. Chemical Composition

#### 2.3.1. Chemical Compositions

Cricket powder was obtained from all three drying processes. Immediately after grinding, a portion of the samples was analyzed for nutritional composition, while the remaining samples were vacuum-sealed in zip-lock bags for storage. The proximate composition of cricket powder subjected to different drying methods was analyzed according to the standard procedures of the Association of Official Analytical Chemists [19]. Protein content was determined using a nitrogen-to-protein conversion factor of N × 6.25 (Method 992.15); also measured were crude fiber (Method 985.29), crude fat (Method 922.06), moisture content (Method 925.10), and ash content (Method 923.03) Then, gross energy was measured using an adiabatic bomb calorimeter (AC500, LECO Corp., St. Joseph, MI, USA). Phosphorus content was assessed using the photometric method (Method 964.06) with an atomic absorption spectrophotometer (T80+, PG Instruments Ltd., Lutterworth, England, UK), while calcium content was analyzed using the titration method (Method 927.02). All analyses were performed in three replicates per sample.

#### 2.3.2. Amino Acid Profiles

The amino acid composition of crickets was carried out at the Scientific Equipment Center, Ubon Ratchathani University, Thailand. Briefly, samples were hydrolyzed with 6 M HCl at 110 °C for 24 h before analysis Then, the hydrolyzed cricket powder samples were analyzed using liquid chromatography tandem mass spectrometry (LC-MS/MS, Model LCMS-8045, Shimadzu Corp., Kyoto, Japan) operated in multiple reaction monitoring mode. Chromatographic separation was performed using an Atlantis^®^ Silica HILIC column (4.6 mm × 100 mm, 3 µm particle size; Waters Corp., Milford, MA, USA). The mobile phases consisted of 5% acetic acid in water (A) and 10% methanol in acetonitrile (B), with a gradient elution at a flow rate of 0.25 mL/min. The injection volume was 2 µL. Quantification was performed using both internal and external standard calibration curves. Amino acid concentrations were measured spectrophotometrically at 570 nm and 440 nm, respectively [20].

### 2.4. Manufacturing Costs

The total drying cost of the three different drying methods was comparatively assessed based on the production cost per kilogram of dried crickets, following the approach described by Alam et al. [21], with some modifications. The cost analysis was conducted using the following equation:

Electrical energy cost (USD) = Power rating (kW) × Drying time (h) × Electricity rate (0.12 USD/kWh)

Labor cost (USD): Unit labor cost was considered as 4.60 USD/day.

Maintenance and depreciation costs (USD): Estimated as a fixed value to represent equipment wear and tear over time, with consideration given to the capital cost and usage frequency of each machine.

The total drying cost was calculated as the sum of electricity, labor, and maintenance and depreciation expenses.

So, cost per kilogram (USD/kg) = Total drying cost (USD) ÷ Final dried weight (kg).

### 2.5. Statistical Analysis

The data were analyzed using the Statistical Analysis System [22]. All data were subjected to analysis of variance with a CRD experimental design. Differences among means with *p* < 0.05 were accepted as representing statistically significant differences. The significance of the differences among the groups was determined by Duncan’s New Multiple Range Test. The statistical model is as follows: Y_ij_ = μ + τ_i_ + ε_ij_, where Y_ij_ = Observation, µ = Overall mean, τ_i_ = Drying method effect (*i* =sun drying, microwave–vacuum, and hot-air oven), ε_ij_ = Experimental error.

## 3. Results

### 3.1. Physical Characteristics

#### 3.1.1. Bulk Density, Angle of Repose, and Color

**Figure 1:**
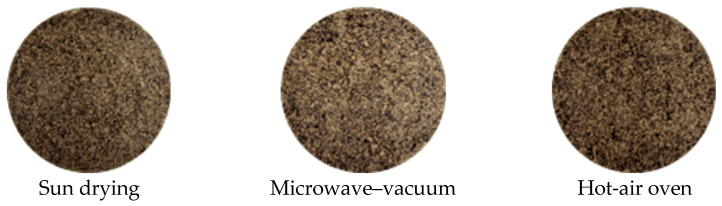
The physical characteristics of cricket powder produced using different drying methods.

The physical properties of cricket powder were influenced by the drying method used, as presented in Table 1 and illustrated in Figure 1. Although not statistically significant (*p* > 0.05), the highest bulk density was observed in samples dried by sun drying, followed by microwave–vacuum-drying and hot-air-oven methods (*p* = 0.06). Similarly, the angle of repose ranged from 43.40° to 45.10°, showing no significant difference among treatments (*p* > 0.05).

In contrast, significant differences were found in color parameters (Table 1 and Figure 1). The L* value, indicating lightness, was significantly higher in microwave–vacuum-dried powder compared to sun drying and hot-air-oven methods (*p* < 0.05). Regarding the a* value (redness), sun-dried samples exhibited significantly higher redness than both hot-air-oven and microwave–vacuum samples (*p* < 0.05). The b* value (yellowness) was also significantly affected, with microwave–vacuum-dried powder having the highest yellowness, followed by sun-drying and hot-air-oven samples (*p* < 0.05). Furthermore, the BI observed in the sun-drying method was significantly higher than that in the microwave and hot-air-oven-drying methods (*p* < 0.05).

#### 3.1.2. Particle Size and Distribution

The particle size and distribution are shown in Table 2. It was found that the particle size distribution of cricket powder was significantly influenced by the drying method, particularly at the coarsest (850 µm) and intermediate (250 µm) sieve sizes. A significant difference was observed in the percentage of particles retained on the 850 µm sieve, where microwave–vacuum drying resulted in the highest retention, followed by sun drying and hot-air-oven methods (*p* < 0.05). Additionally, at the 250 µm size, the microwave–vacuum-drying method showed a significantly lower retention percentage than sun-drying and hot-air-oven treatments (*p* < 0.05). Meanwhile, the other sieve sizes did not show any statistically significant differences (*p* > 0.05).

#### 3.1.3. Microscopic Characterization

**Figure 2:**
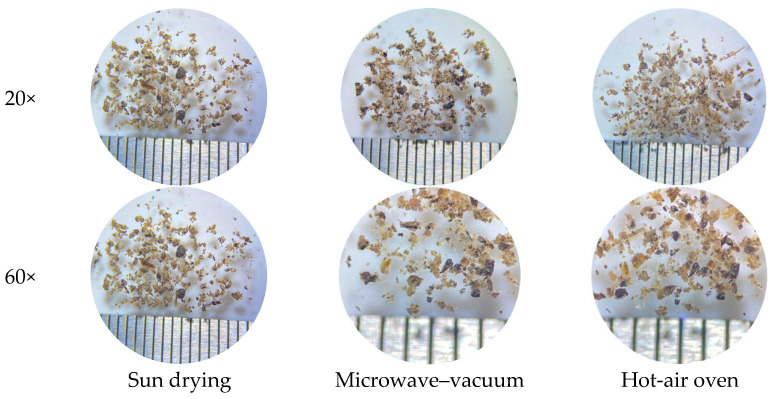
Stereoscopic micrographs of cricket powder produced using different drying methods (sun drying, microwave–vacuum drying, and hot-air-oven drying) at 20× and 60× magnification. The spacing between the scale lines is 1 mm.

Microscopic examination under a stereomicroscope revealed differences in particle morphology and aggregation among the drying methods (Figure 2). At all magnifications (20× and 60×), cricket powder samples exhibited heterogeneous particle structures with visible fragments of varying sizes. The sun-dried powder showed relatively coarse and loosely aggregated particles with irregular shapes. In contrast, the microwave–vacuum-dried sample exhibited denser clusters with more defined edges and a higher degree of particle cohesion. The hot-air-oven-dried sample displayed a more uniform distribution of medium-sized particles, with moderately compact clusters. As shown in Figure 2, the dried crickets powder using sun-drying, microwave–vacuum-drying, and hot-air-oven methods exhibited similar surface characteristics, notably a glossy appearance attributed to the fat coating on the cricket particle.

### 3.2. Chemical Compositions

#### 3.2.1. Chemical Compositions

The effects of different drying method on the nutritional composition of cricket powder are shown in Table 3. Moisture content was highest in the sun-dried sample, followed by the microwave–vacuum-drying and hot-air-oven methods, with all values differing significantly from each other (*p* < 0.05). Gross energy content also varied significantly among treatments (*p* < 0.05). The hot-air-oven method yielded the highest gross energy value, which was significantly greater than that of the microwave–vacuum-dried powder. However, the sun-dried sample had an intermediate value not significantly different from the other two methods. Other nutritional components, including ash, crude protein, ether extract, crude fiber, nitrogen-free extract, calcium, and phosphorus, showed no statistically significant differences (*p* > 0.05) among drying methods.

#### 3.2.2. Amino Acid Profiles

Based on Table 4, which shows the amino acid composition of cricket powder from different drying methods, the amino acid composition of cricket powder was not significantly affected by the drying methods used for all parameters (*p* > 0.05). All essential and non-essential amino acids showed similar concentrations across sun-drying, microwave–vacuum-drying, and hot-air-oven methods.

### 3.3. Manufacturing Costs

Table 5 presents the drying costs of cricket powder using different drying methods with alternative heating sources. The loss associated with each drying method ranged between 61% and 64%. The costs were calculated as the sum of electricity cost, labor cost, and equipment maintenance and depreciation costs. The results for cost per kilogram showed that sun drying yielded the lowest cost at 8.24 USD/kg, followed by microwave–vacuum drying at 8.34 USD/kg. In contrast, hot-air-oven drying incurred the highest cost per kilogram at 40.53 USD/kg.

## 4. Discussion

The post-harvest drying method applied to the crickets plays a critical role in determining the physical and nutritional quality of the resulting powder. Commonly used methods include traditional sun drying, hot-air-oven drying, and modern microwave-assisted vacuum drying, which differ substantially in terms of drying temperature, duration, and oxygen exposure. These differences can significantly influence critical quality parameters, including bulk density, color, particle size, and nutrient retention of the cricket meal [23]. Selecting an appropriate drying method is therefore essential to preserving the nutritional integrity and physical properties of the final product, which are key to its functionality and acceptance as a feed ingredient.

Firstly, the bulk density of feed ingredients refers to the mass per unit volume of a feedstuff in bulk form, which includes the space between particles. It is a crucial property in feed production that impacts storage, handling, and transportation. Several factors affect bulk density, including moisture content (higher moisture increases density), particle size (finer particles pack more densely), particle shape (irregular shapes can increase packing), and processing methods, such as drying and grinding [24]. In the context of insect-based feed ingredients, maintaining appropriate bulk density is crucial for pelleting and feed-blending operations. Meanwhile, the attribute of flowability is one of the most important physical characteristics of feed ingredients, and plays a significant role in its usability in the field. The angle of repose is defined as the maximum slope inclination of any comminuted material when it is barely stable, and it affects the flowability of any granule, including feed [25]. The chemical composition of various ingredients, such as the moisture and fat contents, can also impact the flowability of feed [15,26]. In the present study, the different drying methods had no statistically significant impact on the bulk density or angle of repose of the cricket powder. Although the sun-dried samples exhibited numerically higher bulk density, the difference was not statistically significant, indicating that all three drying methods yielded powders with comparable packing characteristics. The angle of repose, which reflects the flowability of the powder, ranged from 43.40° to 45.10°, with no significant differences observed among treatments. These values fall within the range considered acceptable for granular feed ingredients, aligning with Pornsuwan et al. [14], who reported angles of repose between 41° and 45°, which are classified as indicative of fair to passable flowability. Our results indicate that the cricket powders produced using all drying methods maintain suitable flow characteristics for mixing and processing.

Color variation in cricket powder can have practical implications for its use in animal feed or food products. In formulated feeds, especially for pets or high-value livestock, color and appearance contribute to the perceived quality and consistency of the product. A uniform color is often desirable to ensure that each batch of feed or feed ingredient looks the same; this is a form of quality control. Such variation could also influence acceptance by feed producers, especially in commercial formulations where product aesthetics are considered. If one drying method yields a much darker powder, feed manufacturers might view it as overprocessed or inconsistent in quality [27]. For instance, a dark-brown insect meal could be interpreted as scorched or oxidized, which raises concerns about potential nutrient degradation. Meanwhile, a consistently lighter-colored powder may indicate that it has been processed more gently, which can serve as a quality assurance indicator. Currently, the drying method has a pronounced impact on the color of the cricket powder, as reflected in significant differences in L* (lightness), a* (red-green value), and b* (yellow-blue value). In general, microwave–vacuum-dried cricket powder was markedly lighter in color and less reddish compared to sun-dried and hot-air-oven-dried powder. Specifically, the microwave–vacuum method yielded the highest L* (around 44) and lowest a* (~10) among the three methods, indicating a brighter, less brownish appearance. It also retained slightly more yellowness b* (~9.7) than the other methods. By contrast, both sun drying and oven drying produced darker powders with lower L* values (~41–42). These two methods also led to higher a* values (~12–14), signifying a stronger reddish-brown hue in the powder. The b* values of sun- and oven-dried powders were lower (around 8.4–8.9) than those of the microwave–vacuum-dried sample, suggesting some loss of yellow tone.

High-temperature drying is known to promote a darker appearance in insects. In our data, both sun- and oven-drying methods resulted in browning, with the sun-dried powder exhibiting a slightly redder tone (higher a*). This could be due to the fact that sun drying involves extended exposure to air and sunlight at ambient temperatures; sunlight may stimulate the photo-oxidation of specific molecules. The longer drying time under non-anaerobic conditions likely allows for lipid oxidation to occur, which can contribute to brown discoloration or dulling of color in high-protein feedstuffs [28]. Additionally, a slow drying process can allow for some enzymatic browning (catalyzed by polyphenol oxidases) before the moisture content drops to a sufficient level to inactivate the enzymes [29]. Meanwhile, hot-air-oven drying exposes crickets to elevated temperatures in the presence of oxygen, accelerating Maillard reactions between amino acids and reducing sugars. This non-enzymatic browning produces brown pigmented compounds (melanoidins) that darken the powder and provide red-brown hues [30]. Consequently, oven-dried cricket flour tends to have lower lightness (L*) and higher a*-b* values (more reddish-brown) due to these heat-induced browning reactions. In contrast, microwave–vacuum drying combines rapid internal heating with a low-oxygen environment. The reduced oxygen pressure curtails oxidative browning, and the quick drying process limits the time available for Maillard reactions to progress. Although microwaves do generate heat, the effective temperature can be lower and the exposure time much shorter than in conventional ovens. The result is that less Maillard browning occurs, preserving a higher L* (lighter color) and lower a* (less reddish browning) [23]. Our data confirm an improvement in color parameters with microwave–vacuum drying (brighter, less brown powder) relative to atmospheric drying methods. This aligns with the results of Bawa et al. [23], who found that microwave-dried cricket powder had a superior color (more appealing lightness) compared to oven-dried powder. The trends observed here are consistent with previous studies on the drying of insects and high-protein ingredients. Avoiding intense heat has consistently been shown to preserve a lighter color [14,17]. In addition, the Maillard reaction products formed during the drying process may contribute to higher BI values [17].

The particle size distribution of the cricket powder was significantly influenced by the drying method, with notable differences observed at sieve sizes of 850 µm and 250 µm. Interestingly, microwave–vacuum drying yielded the highest particle retention at 850 µm (41.94%), indicating a coarser particle profile compared to sun drying or hot-air-oven drying. Microwave–vacuum drying operates by volumetrically heating the material, allowing thorough heat penetration while simultaneously removing moisture rapidly under low pressure. In this study, a rotary microwave–vacuum dryer was employed. The dryer continuously rotates the sample container during the drying process, promoting uniform exposure to microwave energy and minimizing hot spots or uneven heating between the interior and edges of the sample. Moreover, the vacuum environment facilitates moisture evaporation at lower temperatures, resulting in rapid drying and the formation of a highly porous, puffed tissue structure. This structural change typically enhances friability, making the dried product more brittle and prone to fracturing, which in turn facilitates the production of finer particles during milling [31,32]. This would typically yield a finer powder, with a larger proportion of particles passing through the 425 µm and 250 µm sieves, at a level similar to, or finer than, oven-dried samples. Indeed, hot-air-oven drying, which involves sustained moderate heating, produced an intermediate particle size distribution. In contrast, sun drying, being slower and often uneven, may result in case hardening of the exoskeleton, where the outer surface dries and hardens before the interior, leaving a dense powder with a fibrous matrix. This tough, compact structure resists fragmentation, yielding larger particles upon milling [33,34].

Although microwave drying is typically associated with internal expansion and structural brittleness that facilitates particle breakage, the present findings suggest that other factors, particularly moisture and fat content, may have contributed to this unexpected outcome. Both moisture and fat act as plasticizing agents, increasing the material’s cohesiveness and resistance to fracture during grinding [35,36]. This likely reduced fragmentation during milling, resulting in larger, less friable particles. Moreover, the higher angle of repose (45.10°) observed in microwave-dried samples, compared to sun-dried (43.40°) and oven-dried (44.70°) powders, supports the hypothesis of greater cohesiveness and reduced flowability.

The data indicate that the choice of drying method significantly influenced the moisture and energy content of the cricket powder, while the protein, fat, fiber, and ash contents remained statistically similar across all treatments. Sun drying resulted in the highest residual moisture (~3.7%), microwave–vacuum drying in the median (~2.4%), and hot-air-oven drying in the lowest (~2.0%). These differences were highly significant; additionally, the gross energy levels also varied. In contrast, crude protein (~55–56% DM) and ether extracts (~26% DM) did not significantly differ between methods. This suggests that, under the conditions tested, intense heat or microwave exposure did not measurably deplete these macro- and micronutrients. Our findings align well with the published data on insect meal and other protein feeds. Crickets (*Acheta domesticus*) are known to yield ~60–70% protein (dry basis) with 10–23% fat [37]. The slightly lower protein (~55%) and higher fat (~26%) levels observed here may reflect the species, diet, or analytical methods used, but are within the expectations for edible insects. Overall, our findings show that the utilized drying method had a minimal impact on the intrinsic protein or fat content of the powder.

However, as mentioned above, the drying method had a great influence on the moisture content of the powder. Moisture differences among treatments have practical implications. A lower residual moisture (as in the hot-air-oven product) effectively concentrates the remaining nutrients on an as-fed basis and greatly improves the shelf life of the pow-der. High moisture levels invite microbial growth and rancidity, so reducing the water activity in the powder is critical [38,39]. Drying insect powders below ~5% moisture achieves a long storage life; modeling predicts ~7 months of ambient stability at 25 °C if the moisture is ~5% [38]. All of our powders (≤3.7% moisture) are well below this threshold, implying even greater stability than this. Lower moisture also simplifies the handling and packaging process (less weight, lower spoilage risk), and can improve processing efficiency. For instance, microwave–vacuum drying removed water in minutes, compared to hours in oven drying, which significantly reduces processing time; however, it may increase operational energy costs and require specialized equipment. In contrast, sun drying is low-cost but weather-dependent, and often takes a much longer time to reach low moisture levels. In summary, the oven-dried cricket meal (~2% moisture) is likely to be the most shelf-stable and concentrated product, whereas the slightly wetter sun-dried powder may be more prone to spoilage if not rapidly processed or consumed.

In terms of amino acid profiles, cricket powders dried by the sun, a microwave-assisted vacuum, or a hot-air oven showed no significant differences in any essential or non-essential amino acids. This is consistent with the previous study of Selaledi and Mabelebele [40], who reported that sun-, oven-, or freeze-dried powder yield essentially identical amino acid profiles. Compared to conventional protein sources, cricket powder offers a more favorable amino acid profile. Insects generally provide higher concentrations of key essential amino acids (Lys 3.28–3.52 and Met 0.67–0.99 g/100g) than soybean (Lys 3.08 and Met 0.71 g/100g) or other plant meals [8,41]. This suggests that cricket protein can, at the least, rival these traditional feeds in nutritional quality [42]. In practice, cricket meal contains all the essential amino acids at levels that meet or exceed the ideal protein profile for poultry, similar to fishmeal [9,43]. The net effect is that cricket powder could replace a portion of soybean or fishmeal in diets without causing amino acid deficiencies. The relatively low methionine content, typical of insects, generally necessitates synthetic supplementation when formulating commercial feeds. Indeed, previous reviews highlight that insect meals contain an abundant supply of lysine and threonine, making them excellent complements to cereal-based diets [10,11].

In terms of drying cost evaluation (Table 5), based on the method of Alam et al. [21], microwave–vacuum drying showed a lower cost per kilogram in small-scale processing (8.34 USD/kg); however, this method may not be the most economical option for large-scale operations. Considering the capacity of the hot-air oven (with a maximum loading of 300 kg) and the non-electric nature of solar drying, both sun drying and hot-air-oven drying offer greater scalability. Their unit costs tend to remain relatively stable, or even decrease slightly as the batch size increases, due to fixed electricity and labor costs. In contrast, microwave–vacuum drying has a limited batch capacity of only 3 kg per cycle. Therefore, processing a total of 300 kg of raw material would require 100 drying cycles, each with its own associated labor, electricity, and maintenance costs. This results in a total drying cost of approximately USD 933.45 or USD 8.34 per kilogram of dried weight. While the cost per kilogram remains constant under linear scaling, the cumulative operational workload, time requirement, and equipment wear become significant drawbacks for high-volume processing. These factors highlight the practical limitations of microwave–vacuum drying for large-scale commercial applications despite its cost efficiency at a small scale.

Based on the calculated cost per kilogram of dried crickets, the lower cost of sun drying can be attributed to its minimal energy consumption, as it does not require electricity. However, it is also the most time-consuming, requiring 48 h of exposure to fluctuating weather conditions, which may pose challenges in terms of consistency and microbial safety [33]. In contrast, microwave–vacuum drying, although rapid (9 min), involves higher initial and operational costs, including elevated labor, energy, and equipment maintenance expenses. These findings are consistent with those of Teleken et al. [32], who reported that microwave systems, although efficient in moisture removal and nutrient retention, are typically associated with higher capital and operating costs. Hot-air-oven drying represents a balanced approach, offering a reasonable drying time (48 h), moderate costs, and reliable control over drying conditions, making it suitable for semi-industrial applications. The consistent thermal environment supports efficient moisture removal, which may contribute to improved energy utilization and product stability [31]. From an economic standpoint, the cost per kilogram is a key metric for feed manufacturers, particularly in developing economies where production costs must be minimized. Integrating such cost analyses into production decision-making aligns with the recommendations by Makkar et al. [44], who emphasized the importance of both nutritional value and cost-efficiency in evaluating novel protein sources for animal feed. In summary, the choice of drying process should be aligned with the production scale, resource availability, and target market. While microwave–vacuum drying offers technical advantages, sun drying and hot-air-oven drying provide more economically viable options for small- to medium-scale producers.

In summary, all drying methods produced cricket powder with nutrient contents comparable to those of other high-quality insect meals reported in the literature. No method excessively degraded the crickets’ protein or fat content, and the resultant powders offer favorable macronutrient profiles for poultry feed. Hot-air drying achieved the driest, most energy-rich powder, while sun and microwave methods produced equally protein-rich meals with a slightly higher moisture level. The considered selection of a drying technique can therefore prioritize efficiency or cost without compromising nutritional quality, supporting the use of cricket meal as a sustainable feed ingredient, consistent with the previously published evidence [21].

## 5. Conclusions

In conclusion, although drying methods influenced physical characteristics such as bulk density, color, angle of repose, and particle distribution, they did not significantly alter the nutritional composition or amino acid profile of cricket powder. All methods preserved the nutritional integrity of the product, confirming its potential as a sustainable protein source in poultry feed. Based on the overall experimental data, including physical flow properties and nutritional value, hot-air-oven drying appeared to be the most suitable method, offering the lowest moisture content without adverse effects on physical properties or nutritional composition, which are advantageous for storage stability and feed formulation. Furthermore, this method offers a practical balance between drying efficiency and operational cost, making it a practical choice for medium- to large-scale production of cricket powder where quality consistency and operational feasibility are priorities.

## Figures and Tables

**Table 1:** Physical characteristics of cricket powder from different drying methods.

Parameters	Drying Methods			SEM	*p*-Value
	Sun Drying	Microwave–Vacuum	Hot-Air Oven		
Bulk density (g/L)	462.35	453.89	443.30	12.29	0.06
Angle of repose (^o^)	43.40	45.10	44.70	0.87	0.12
Color					
L*	41.98 ^b^	44.07 ^a^	41.77 ^b^	0.59	<0.01
a*	14.20 ^a^	10.38 ^b^	12.73 ^a^	0.96	<0.01
b*	8.91 ^b^	9.66 ^a^	8.45 ^b^	0.42	0.01
Browning index (BI)	47.51 ^a^	41.36 ^b^	44.00 ^b^	1.74	<0.01

^a,b^ Means within rows with difference superscript letters differ significantly at *p* < 0.05. L* = lightness; a* = redness; b* = yellowness. SEM: Standard error of mean.

**Table 2:** Particle size and distribution of cricket powder from different drying methods ^1^.

Sieve Mesh No.	Sieve Size (µm)	Sample (g)			Retain (%)			SEM	*p*-Value	Cumulative (%)			Passing (%)		
		Sun Drying	Microwave–Vacuum	Hot-Air Oven	Sun Drying	Microwave–Vacuum	Hot-Air Oven			Sun Drying	Microwave–Vacuum	Hot-Air Oven	Sun Drying	Microwave–Vacuum	Hot-Air Oven
20	850	37.46	42.02	35.94	37.41 ^ab^	41.94 ^a^	35.90 ^b^	2.45	0.04	37.41	41.94	35.90	62.59	58.06	64.10
40	425	55.64	52.62	57.08	55.56	52.53	57.01	1.89	0.06	92.97	94.47	92.91	7.03	5.53	7.09
60	250	6.00	4.68	6.04	5.99 ^a^	4.67 ^b^	6.03 ^a^	0.51	0.02	98.96	99.14	98.94	1.04	0.86	1.06
80	180	1.04	0.86	1.06	1.04	0.86	1.06	0.26	0.64	100.00	100.00	100.00	0.00	0.00	0.00
100	150	0.00	0.00	0.00	0.00	0.00	0.00	-	-	100.00	100.00	100.00	0.00	0.00	0.00
Pan		0.00	0.00	0.00	0.00	0.00	0.00	-	-	100.00	100.00	100.00	0.00	0.00	0.00
Total		100.14	100.18	100.12	100.00	100.00	100.00								

^1^ The samples were analyzed in 10 replicates per drying method. ^a,b^ Means within rows with difference superscript letters differ at *p* < 0.05. SEM: Standard error of mean.

**Table 3:** Chemical composition of cricket powder (g/100 g dry basis) from different drying methods.

Parameters	Drying Methods			SEM	*p*-Value
	Sun Drying	Microwave–Vacuum	Hot-Air Oven		
Moisture (%)	3.73 ^a^	2.37 ^b^	1.99 ^c^	0.05	<0.01
Ash (%)	5.24	4.61	4.81	0.44	0.39
Crude protein (%)	55.82	54.99	54.47	0.08	0.11
Ether extract (%)	25.60	26.80	26.87	0.07	0.08
Crude fiber (%)	9.24	9.32	9.21	0.16	0.51
Nitrogen-free extract (%)	4.10	4.28	4.74	0.16	0.29
Calcium (%)	0.11	0.19	0.10	0.04	0.23
Phosphorus (%)	0.01	0.01	0.01	0.00	0.16
Gross energy (kcal/kg)	5897.44 ^b^	5966.72 ^ab^	6126.43 ^a^	123.90	0.04

^a–c^ Means within rows with difference superscript letters differ at *p* < 0.05. SEM: Standard error of mean.

**Table 4:** Amino acid composition of cricket powder from different drying methods.

Parameters	Drying Methods			SEM	*p*-Value
	Sun Drying	Microwave–Vacuum	Hot-Air Oven		
Essential amino acids (g/100g)					
Lysine	3.28	3.49	3.52	0.44	0.77
Valine	2.21	2.60	2.61	0.51	0.57
Leucine	2.20	2.24	2.24	0.23	0.97
Methionine	0.67	0.93	0.88	0.40	0.70
Isoleucine	1.09	1.21	1.22	0.21	0.71
Histidine	0.04	0.08	0.06	0.03	0.42
Threonine	1.03	1.14	1.12	0.13	0.58
Phenylalanine	1.32	1.72	1.68	0.60	0.69
Tryptophan	0.35	0.40	0.41	0.20	0.93
Total essential amino acids	12.19	13.81	13.74	1.91	0.73
Non-essential amino acids (g/100g)					
Glutamic	5.35	5.55	5.57	0.48	0.87
Glutamine	1.53	1.82	1.82	0.75	0.90
Aspartic	4.30	4.34	4.31	0.32	0.99
Alanine	2.65	2.85	2.84	0.79	0.94
Glycine	1.49	1.58	1.58	0.58	0.98
Proline	2.85	2.75	2.86	0.82	0.98
Serine	1.34	1.28	1.36	0.33	0.95
Arginine	2.70	2.68	2.72	0.32	0.99
Tyrosine	2.22	2.24	2.24	0.58	0.78
Cysteine	0.41	0.41	0.43	0.77	0.67
Total non-essential amino acids	24.84	25.50	25.73	3.69	0.98

SEM: Standard error of mean.

**Table 5:** Evaluation of overall drying cost for cricket powder from different drying methods.

Parameters	Drying Methods		
	Sun Drying	Microwave–Vacuum	Hot-Air Oven
Batch size (kg)	3.00	3.00	3.00
Drying time (h)	48.00	0.25	48.00
Total dried output (kg/batch)	1.15	1.12	1.09
Loss rate (%)	61.67	62.67	63.67
Electricity cost (USD/kWh)	0.12	0.12	0.12
Power rating (kWh)	0.00	0.27	0.17
Electricity cost (USD)	0.00	0.16	33.45
Labor cost (USD)	9.17	4.59	9.17
Equipment maintenance cost (USD/batch)	0.31	4.59	1.52
Total cost (USD/batch)	9.48	9.34	44.17
Cost per dried weight (USD/kg)	8.24	8.34	40.53
Total cost per 300 kg of fresh cricket (USD)	14.22	933.58	66.26

## Data Availability

The original contributions presented in this study are included in the article. Further inquiries can be directed to the corresponding author.
